# Multifaceted role of FAM210B in hepatocellular carcinoma: Implications for tumour progression, microenvironment modulation and therapeutic selection

**DOI:** 10.1111/jcmm.70031

**Published:** 2024-08-28

**Authors:** Xianzhu Pan, Jun Xu, Yuanqin Zhou

**Affiliations:** ^1^ Department of Pathology and Pathophysiology, School of Basic Medicine Anhui Medical College Hefei China

**Keywords:** drug sensitivity, FAM210B, hepatocellular carcinoma, immunotherapy, tumour microenvironment

## Abstract

Hepatocellular carcinoma (HCC) is a common and lethal liver cancer characterized by complex aetiology and limited treatment options. FAM210B, implicated in various cancers, is noteworthy for its potential role in the progression and treatment response of HCC. Yet, its expression patterns, functional impacts and correlations with patient outcomes and resistance to therapy are not well understood. We employed a comprehensive methodology to explore the role of FAM210B in HCC, analysing its expression across cancers, subcellular localization and impacts on cell proliferation, invasion, migration, biological enrichment and the immune microenvironment. Additionally, we investigated its expression in single cells, drug sensitivity and relationships with genomic instability, immunotherapy efficacy and key immune checkpoints. While FAM210B expression varied across cancers, there was no notable difference between HCC and normal tissues. Elevated levels of FAM210B were associated with improved survival outcomes. Subcellular analysis located FAM210B in the plasma membrane and cytosol. FAM210B was generally downregulated in HCC, and its suppression significantly enhanced cell proliferation, invasion and migration. Biological enrichment analysis linked FAM210B to metabolic and immune response pathways. Moreover, its expression modified the immune microenvironment of HCC, affecting drug responsiveness and immunotherapy outcomes. High expression levels of FAM202B correlated with increased resistance to sunitinib and enhanced responsiveness to immunotherapy, as evidenced by associations with tumour mutation burden, PDCD1, CTLA4 and TIDE scores. FAM210B exerts a complex influence on HCC, affecting tumour cell behaviour, metabolic pathways, the immune microenvironment and responses to therapy.

## 1 INTRODUCTION

Hepatocellular carcinoma (HCC) is the predominant form of primary liver cancer and a leading cause of cancer‐related mortality worldwide.^1^, ^2^ It commonly arises in individuals with chronic liver conditions such as cirrhosis, often due to hepatitis B or C virus infections, alcoholic liver disease or non‐alcoholic fatty liver disease (NAFLD).^3^ The development of HCC involves a complex interplay of genetic, epigenetic and environmental factors that promote liver cell malignancy.^4^, ^5^ Early symptoms are typically absent, complicating early detection efforts.^6^ Treatment varies, ranging from curative approaches like surgery and liver transplantation in the early stages to palliative treatments such as systemic therapies and transarterial chemoembolization in advanced stages.^7^, ^8^, ^9^ Despite these options, the prognosis for HCC patients remains dismal, particularly when diagnosed late,^10^ highlighting the critical need for enhanced surveillance and early detection in susceptible populations, along with continuous research into new therapeutic targets and strategies.^11^ The molecular intricacies of HCC progression and treatment response involve a wide array of genetic and epigenetic alterations, signalling pathway disruptions and interactions within the tumour microenvironment.^12^, ^13^ The FAM210B gene, implicated in diverse cancer types, has recently gained attention due to its potential role in cancer biology.^14^

FAM210B is expressed variably across cancers and has been linked to several key biological processes.^14^, ^15^ Studies have shown that FAM210B, a target of erythropoietin, is essential for erythroid heme production by regulating mitochondrial iron uptake and ferrochelatase activity.^16^ Additionally, research indicates that the loss of FAM210B, a mitochondrial protein, promotes metastasis in ovarian cancer through metabolic reprogramming driven by PDK4 dependency.^17^ However, the specific mechanisms by which FAM210B influences HCC, as well as its potential as a therapeutic target and biomarker for treatment response, remain to be fully explored.

This study aims to address these gaps by providing a comprehensive analysis of FAM210B's role in HCC through various methods, including pan‐cancer expression analysis, subcellular localization studies, functional assays in HCC cell lines and assessments of the immune microenvironment and drug sensitivity. Additionally, we investigate the correlation between FAM210B expression and crucial clinical outcomes, such as survival, drug resistance and responsiveness to immunotherapy. Understanding FAM210B's role in HCC could reveal critical insights into the disease's molecular mechanisms and lead to the identification of novel therapeutic targets and biomarkers for personalized treatment approaches.

## 2 METHODS

### 2.1 Public data acquisition

Clinical and transcriptional data for HCC were sourced from The Cancer Genome Atlas (TCGA) database, and retrieved through the TCGA‐GDC portal using the R package TCGAbiolinks (version 2.18.0).^18^ Transcription data, formatted as STAR‐Counts, were converted to transcripts per kilobase million (TPM) values using custom R scripts. Clinical data, initially formatted in bcr‐xml, along with mRNA and lncRNA differentiation, were downloaded from the ENSEMBLE website. To enhance the data quality, a pre‐processing step was conducted using the edgeR package (version 3.32.1). Data on tumour mutational burden (TMB) and microsatellite instability (MSI) were obtained from the Researcher's Home portal, while tumour stemness indices (mRNAsi and EREG‐mRNAsi) were derived from prior studies.^19^ Representative immunohistochemistry images were sourced from the Human Protein Atlas (HPA) database.^20^ Biological enrichment analysis was conducted using the Gene Set Enrichment Analysis (GSEA) algorithm.^21^

### 2.2 Tissue collection

This research aimed to elucidate FAM210B's role in HCC pathology. Tissue specimens, including cancerous and adjacent non‐cancerous liver tissues, were collected from consenting patients at the Second Affiliated Hospital of Anhui Medical University, following ethical guidelines approved by the Institutional Review Board.

### 2.3 Haematoxylin and eosin (HE) staining and FAM210B immunohistochemistry (IHC)

HE staining was performed to evaluate the histopathological characteristics of the liver tissues. Paraffin‐embedded tissue sections were deparaffinized, rehydrated and stained with haematoxylin for 5 min, followed by eosin for 2 min. The stained sections were then dehydrated, cleared and mounted for microscopic examination. For FAM210B immunohistochemistry (IHC), tissue sections were deparaffinized and rehydrated. Antigen retrieval was conducted by heating the sections in a citrate buffer (pH 6.0) for 15 min. After cooling to room temperature, endogenous peroxidase activity was blocked using 3% hydrogen peroxide for 10 min. The sections were then incubated with a primary antibody against FAM210B (Novus, NBP2‐14523; 1:200 dilution) at 4°C overnight. Following this, the sections were washed and incubated with a biotinylated secondary antibody for 30 min at room temperature, followed by streptavidin‐HRP for another 30 min. The immunoreactivity was visualized using diaminobenzidine as the chromogen and counterstained with haematoxylin. The stained sections were examined under a microscope to assess FAM210B expression in HCC and adjacent non‐cancerous tissues. The intensity and distribution of FAM210B staining were evaluated, with positive staining indicated by a brown colour in the cytoplasm and membrane of the cells.

### 2.4 RNA extraction and detection

RNA was extracted using the RNeasy Mini Kit (Qiagen), following the manufacturer's protocol to ensure high‐quality RNA. Quantification of FAM210B mRNA levels was performed via quantitative real‐time PCR (qRT‐PCR), with GAPDH as the normalization reference (SYBR green system, vazyme). The primers used were: FAM210B‐forward: 5’‐TCGTTCGCTCAAGCTTGTATCTA‐3′; FAM210B‐reverse: 5′‐TATGGTTGTTCACCTCTCGTTCAC‐3′; GAPDH‐forward: 5′‐AATGGCAGCAGGCACAAGTACC‐3′; and GAPDH‐reverse: 5′‐CAAGGGCACAGAGACTAGCGTAATG‐3′.

### 2.5 Cell culture and gene knockdown

HCC cell lines (Hep3B and HepG2) and normal liver cell lines were propagated under optimal conditions purchased from the Chinese Academy of Sciences Cell Bank, with media enriched with 10% foetal bovine serum to support cell growth and viability. To investigate FAM210B's functional role, we employed shRNA to specifically knock down FAM210B expression, contrasting the effects with cells transfected with non‐targeting shRNA as controls. The shRNA sequences that targeted FAM210B were: shRNA#1 sense, 5′‐AACAGCTTGTGGATTGCATAGGCCA‐3′ and antisense: 5′‐TGGCCTATGCAATCCACAAGCTGTT‐3′; shRNA#2 sense, 5′‐TTCTCACTGGCGCAAACAGCTTGTG‐3′ and antisense, 5′‐CACAAGCTGTTTGCGCCAGTGAGAA‐3′.

### 2.6 CCK‐8 assay

Cell proliferation was evaluated using the Cell Counting Kit‐8 (CCK‐8, Beyotime). In this study, cells were seeded in a 96‐well plate at a density of 1 × 10^4^ cells per well. 10 μL of CCK‐8 solution was added to each well at predetermined time points (0, 24, 48, 72 h), followed by incubation for 2 h. Next, the OD value was measured at a wavelength of 450 nm using a microplate reader, reflecting cell viability and quantity.

### 2.7 Colony formation assay

The colony formation assay evaluates the proliferative capacity of cells. Cells were seeded at 500 cells per well in a six‐well plate and cultured at 37°C in a 5% CO_2_ atmosphere for approximately 2 weeks until visible colonies formed. After culture, the medium was removed, cells were washed with PBS, fixed with 4% formaldehyde for 30 min, and stained with 0.1% crystal violet for 15 min. Finally, colonies were rinsed with water, dried and counted under a microscope.

### 2.8 Transwell assay

The transwell assay assesses cell migration and invasion capabilities. In this study, a transwell apparatus with an 8‐μm pore size membrane was used (Corning). For migration assays, cell suspensions were added to the upper chamber of the transwell, while the lower chamber contained FBS. For invasion assays, the upper chamber membrane was pre‐coated with Matrigel to simulate the extracellular matrix, before adding cell suspensions. After 24 h, non‐migrated or non‐invaded cells in the upper chamber were removed with a cotton swab. Cells that had migrated or invaded to the lower side of the membrane were fixed with 4% formaldehyde, stained with 0.1% crystal violet and counted under a microscope.

### 2.9 Immune microenvironment and drug sensitivity

The assessment of the immune microenvironment and drug sensitivity concerning FAM210B expression involved the application of computational algorithms to estimate the infiltration of various immune cell types, including CIBERSORT, EPIC, MCPCOUNTER, TIMER and Xcell.^22^, ^23^, ^24^, ^25^, ^26^ Furthermore, information on drug sensitivity was collected from the Genomics of Drug Sensitivity in Cancer (GDSC) database.^25^

### 2.10 Construction of prognosis model

Initially, FAM210B‐associated molecules were identified using correlation analysis. Univariate Cox regression was conducted to determine survival‐related molecules, while LASSO regression, a machine learning algorithm, was applied to minimize variable redundancy, ensuring only the most significant predictors were included in the model. The final model was developed using multivariate Cox regression, incorporating selected variables to compute a risk score that categorizes patients into distinct risk groups based on their predicted survival outcomes.

### 2.11 Statistical analysis

Statistical analyses were conducted using SPSS software (Version 22.0, IBM Corp., Armonk, NY, USA) and the R programming environment (Version 4.1.2, R Foundation for Statistical Computing, Vienna, Austria). The Kaplan–Meier (KM) method was utilized for estimating survival curves, and the log‐rank test was used to compare survival distributions. For all statistical tests, a two‐tailed approach was adopted, and a *p*‐value below 0.05 was considered statistically significant. Corrections for multiple comparisons were applied using the false discovery rate method where appropriate to ensure the reliability of the results.

## 3 RESULTS

### 3.1 Expression level of FAM210B in HCC

**FIGURE 1:**
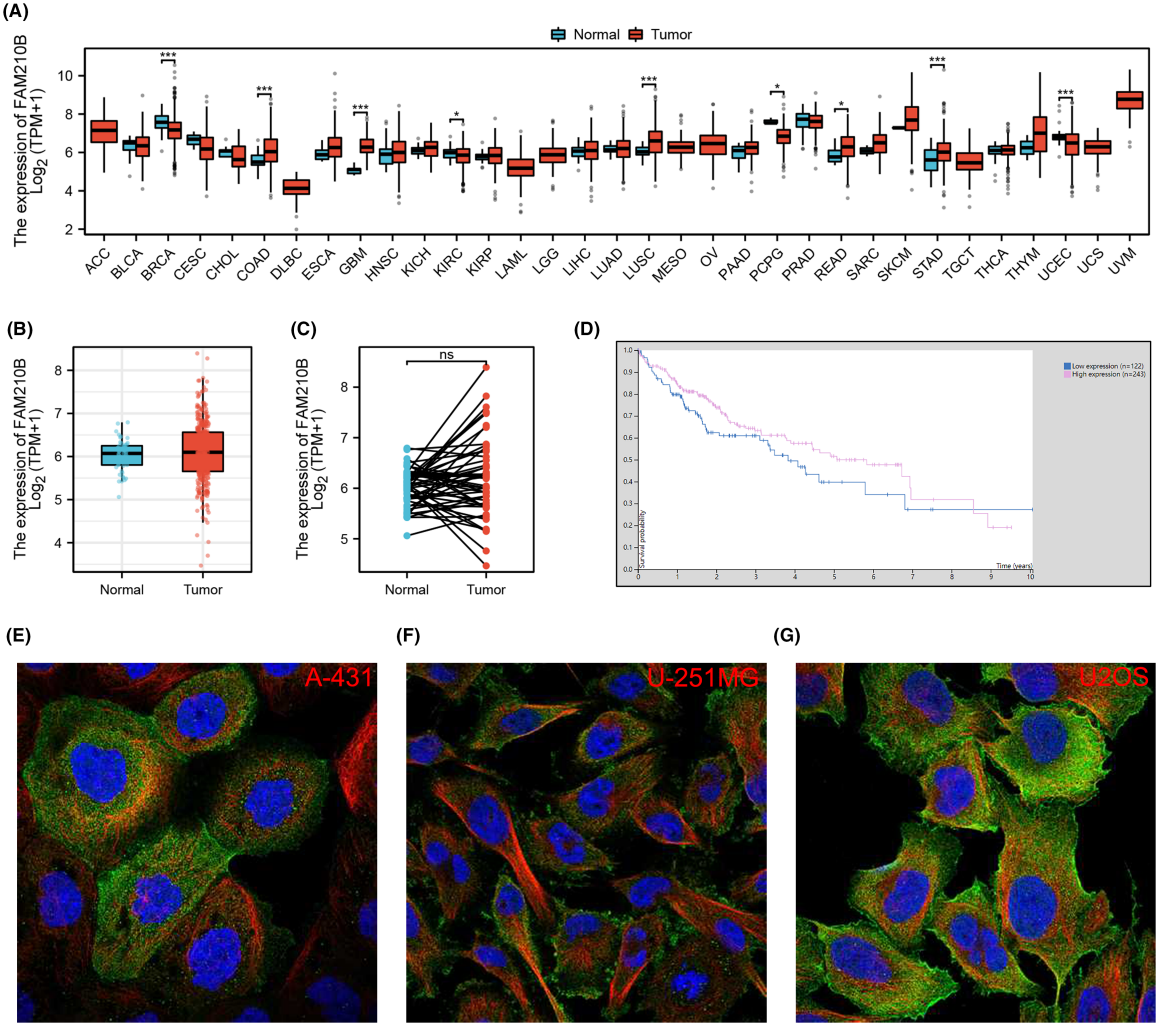
The expression level of FAM210B in HCC. (A) The expression level of FAM210B in pan‐cancer; (B, C) The expression level of FAM210B in paired and non‐paired HCC tissue; (D) KM survival curves of FAM210B in HCC; (E–G): The subcellular location of FAM210B in specific cells from HPA database in difference cell lines.

The flowchart of whole study was shown in Figure S1. We initiated our investigation by examining FAM210B's expression across various cancers. Our analysis revealed that FAM210B exhibited differential expression at the RNA level in numerous cancers, including breast, colon, glioma, renal and lung cancers (Figure 1A). However, in the context of HCC, FAM210B did not show a significant difference in expression when compared to normal tissue, regardless of whether the samples were paired or non‐paired (Figure 1B,C). Interestingly, KM survival curves suggested that patients with higher levels of FAM210B might experience better survival outcomes (Figure 1D). Further exploration into FAM210B's subcellular localization using the HPA database indicated its predominant presence in the plasma membrane and cytosol (Figure 1E–G).

### 3.2 FAM210B was downregulated and inhibits the proliferation, invasion and migration ability of HCC cells

**FIGURE 2:**
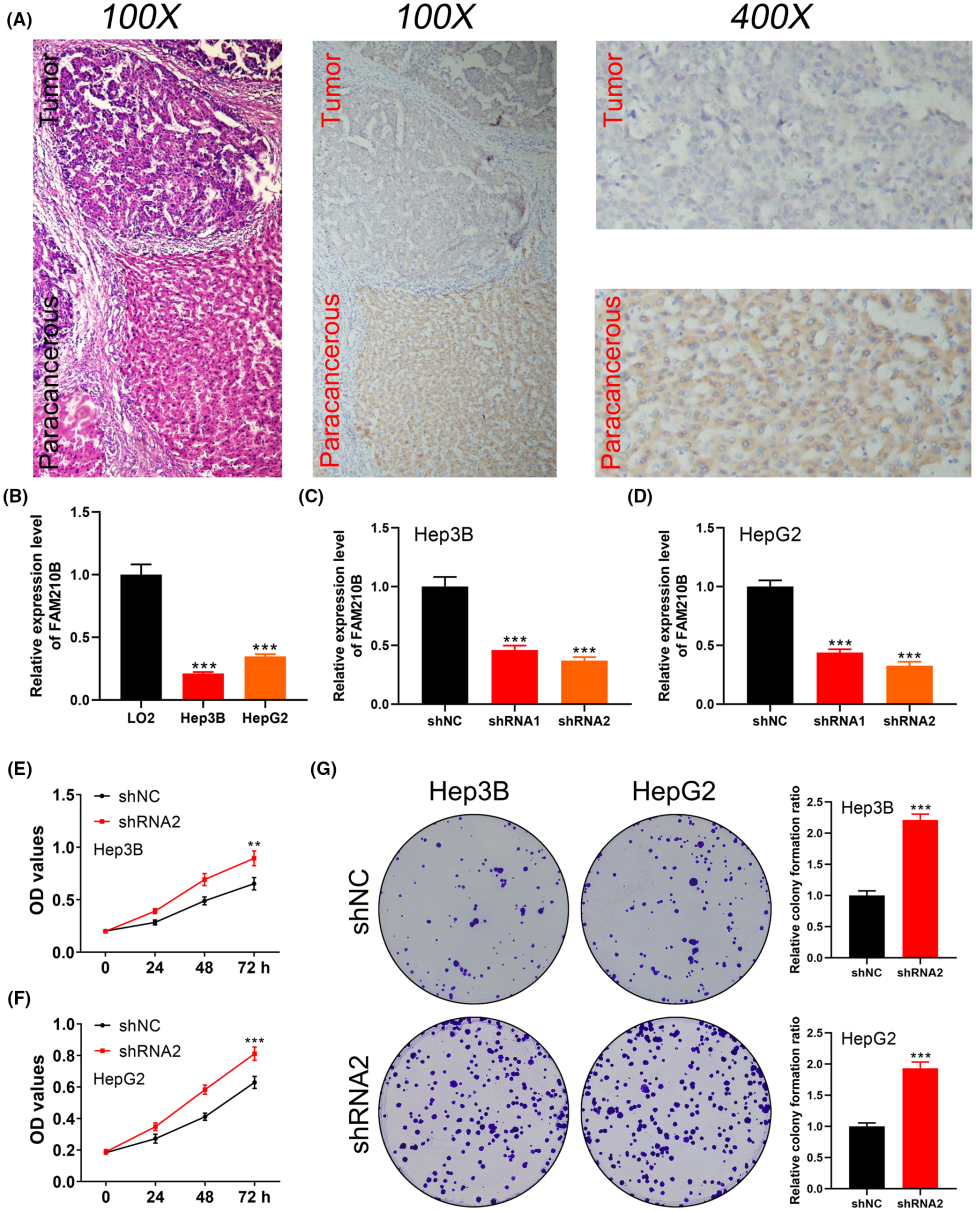
FAM210B was downregulated and inhibit the proliferation ability in HCC cells. (A) The protein level of FAM210B in HCC and normal tissue (the left panel = HE staining); (B) FAM210B was downregulated in HCC cells compared with the normal cells; (C, D) QRT‐PCR was used to detect the knockdown efficiency of FAM210B in HCC cells; (E, F) CCK8 assay was used to the proliferation ability of FAM210B knockdown and control cells; (G) Colony formation assay was used to the proliferation ability of FAM210B knockdown and control cells.

**FIGURE 3:**
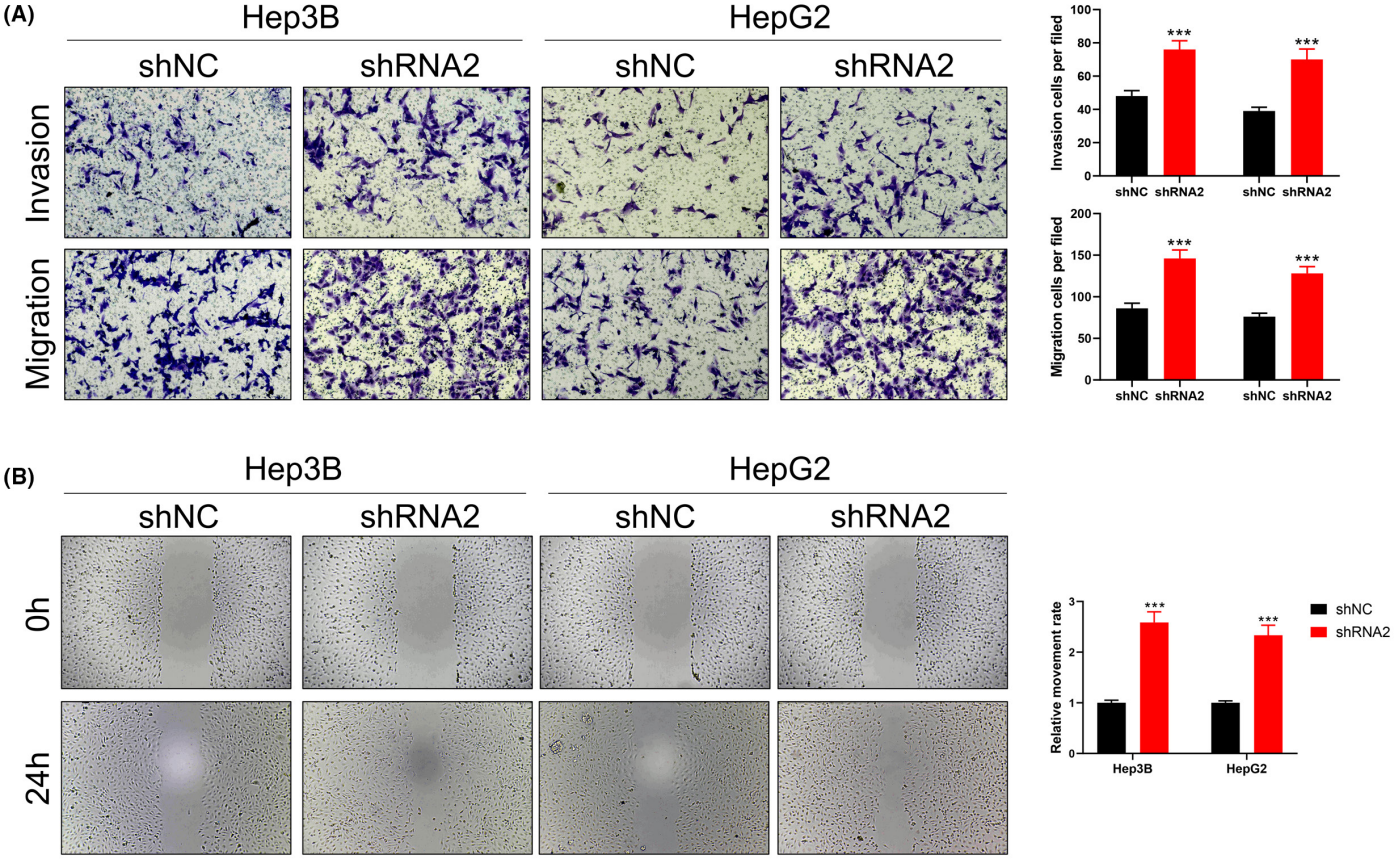
FAM210B suppress the invasion and migration ability of HCC cells. (A) Transwell assay was performed in FAM210B knockdown and control cells; (B) Wound‐healing assay was performed in FAM210B knockdown and control cells.

Subsequent investigations focused on the protein level of FAM210B in HCC versus adjacent tissue, revealing a lower protein level in HCC tissues (Figure 2A). qRT‐PCR analysis confirmed FAM210B's downregulation at the mRNA level in HCC cells compared to normal LO2 cells (Figure 2B). By knocking down FAM210B, we aimed to decipher its biological role in HCC cells (Figure 2C,D). Our findings from CCK8 and colony formation assays indicated that FAM210B inhibition significantly promotes HCC cell proliferation (Figure 2E–G). Moreover, transwell assays demonstrated that FAM210B knockdown markedly enhances the invasion and migration capabilities of HCC cells (Figure 3A,B).

### 3.3 Biological enrichment of FAM210B in HCC

**FIGURE 4:**
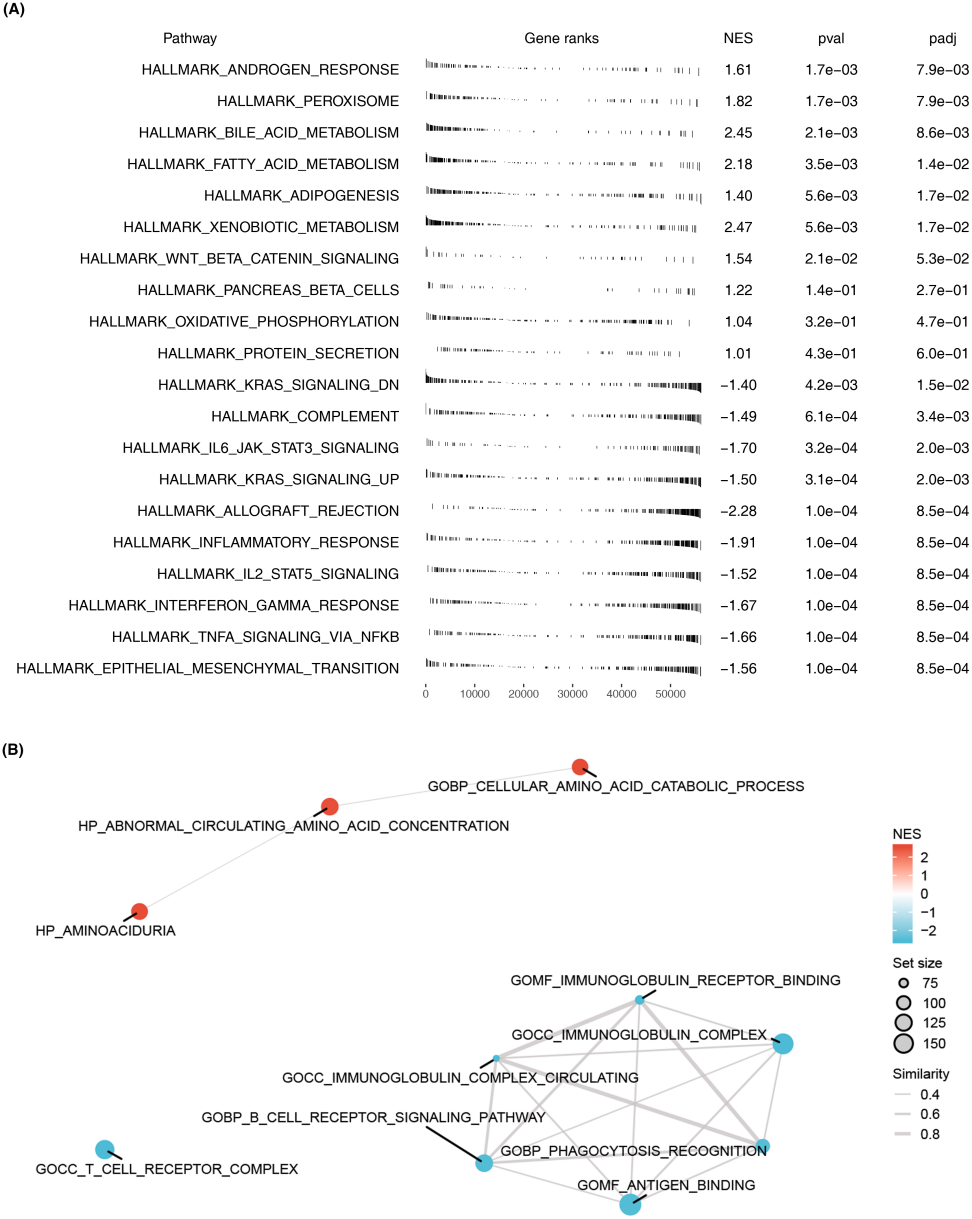
Biological role of FAM210B in HCC. (A) GSEA analysis of FAM210B based on Hallmark gene set; (B) GSEA analysis of FAM210B based on GO gene set.

Our biological enrichment analysis of FAM210B in HCC through GSEA revealed that patients with high FAM210B levels showed significant upregulation in terms related to androgen response, peroxisome, bile acid, fatty acid and xenobiotic metabolism. Conversely, terms associated with epithelial‐mesenchymal transition (EMT), interferon‐gamma response, IL2/STAT5 signalling and inflammatory were notably downregulated (Figure 4A). GSEA analysis based on the GO gene set indicated that in the patients with high FAM210B level, the terms of aminoaciduria, abnormal circulating amino acid concentration and cellular amino acid catabolic process were upregulated, while the terms of T‐cell receptor complex, B‐cell receptor signalling pathway, immunoglobulin complex circulating, phagocytosis recognition, antigen binding, immunoglobulin complex and immunoglobulin receptor binding were downregulated (Figure 4B).

### 3.4 Influence of FAM210B on the HCC microenvironment

**FIGURE 5:**
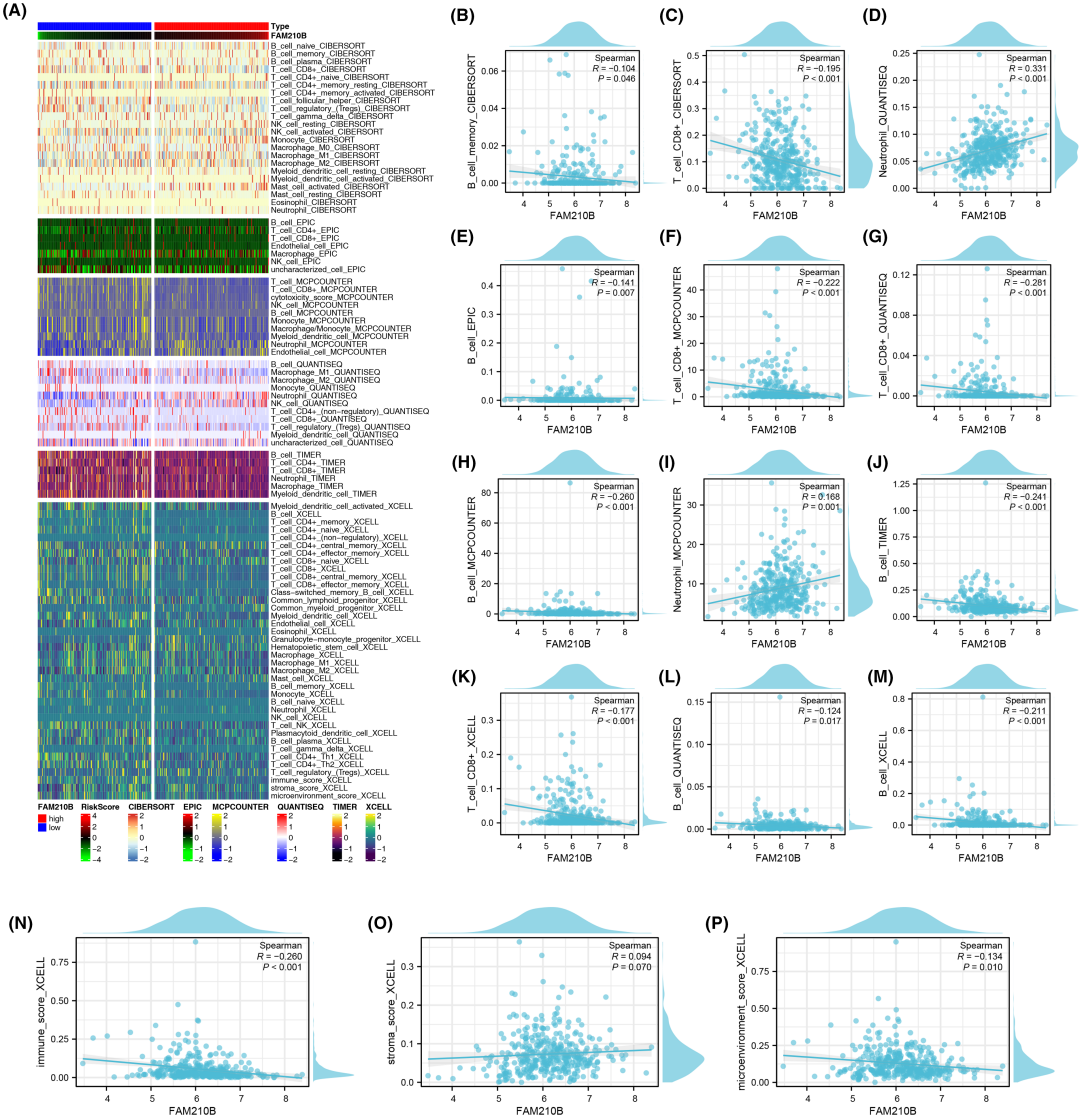
Influence of FAM210B on HCC microenvironment. (A) Immune cells was quantified using multiple algorithms; (B–M) Correlation between FAM210B and specific cells; (N–P) Correlation between FAM210B and microenvironment score.

Furthermore, we quantified the immune microenvironment of HCC based on multiple algorithms (Figure 5A). Quantification of the HCC immune microenvironment exposed a positive correlation between FAM210B and neutrophils, contrasting with negative correlations with B cells, memory B cells and CD8+ T cells (Figure 5B–M). Additionally, FAM210B showed a positive association with the stromal score, yet negative associations with the immune score and the overall microenvironment score, highlighting its complex role in modulating the HCC microenvironment (Figure 5N–P).

### 3.5 Single‐cell analysis and drug sensitivity

**FIGURE 6:**
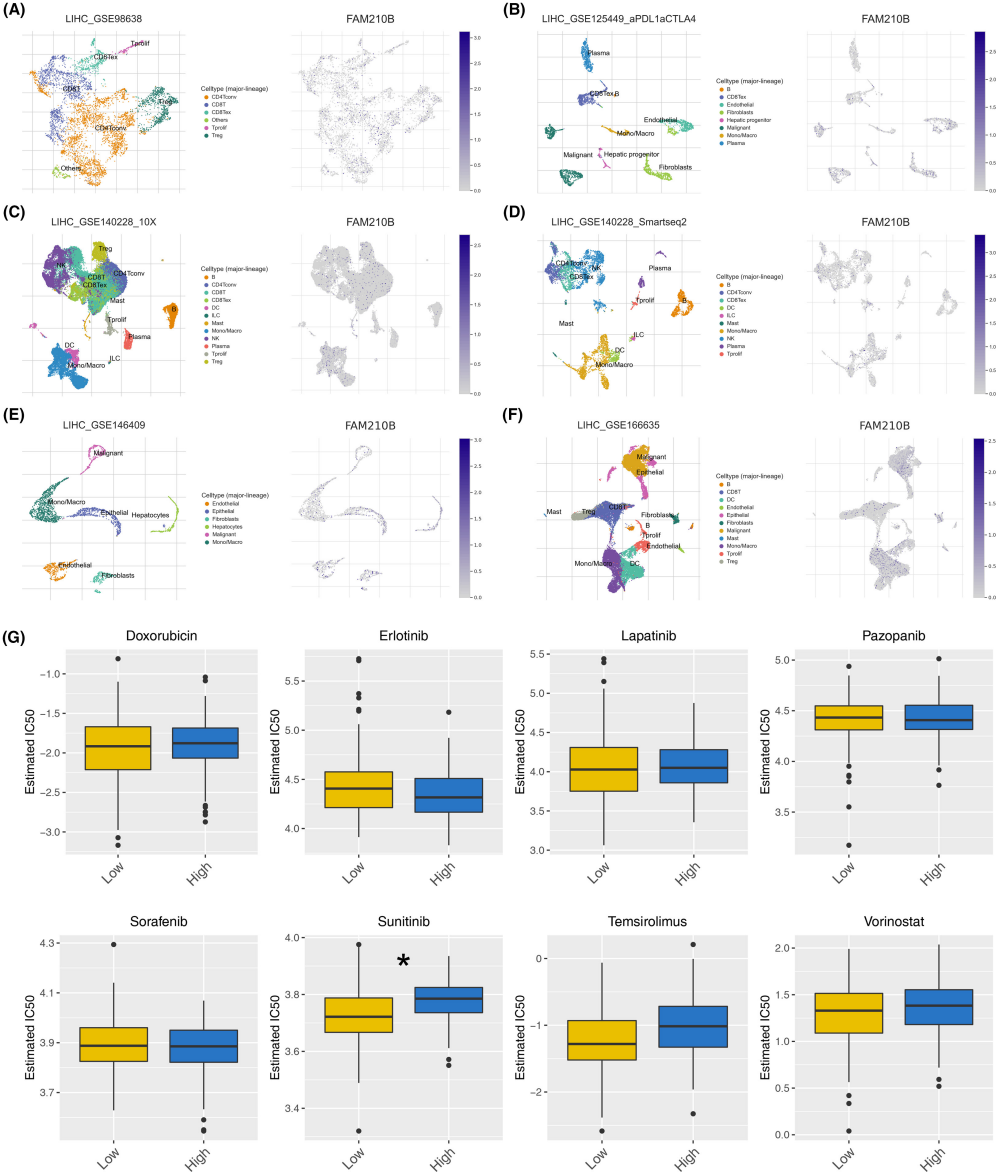
Single‐cell and drug sensitivity analysis. (A–F) The single‐cell analysis of FAM210B in HCC microenvironment; (G) IC50 of specific drugs in cells with high and low FAM210B expression.

Single‐cell analysis, leveraging data from the TISCH project, revealed a uniform distribution of FAM210B across various cell types (Figure 6A–F). Drug sensitivity analysis suggested that high FAM210B expression could be linked to increased resistance to sunitinib, indicating potential implications for treatment strategies (Figure 6G).

### 3.6 FAM210 was associated with the mutation and immunotherapy response of HCC

**FIGURE 7:**
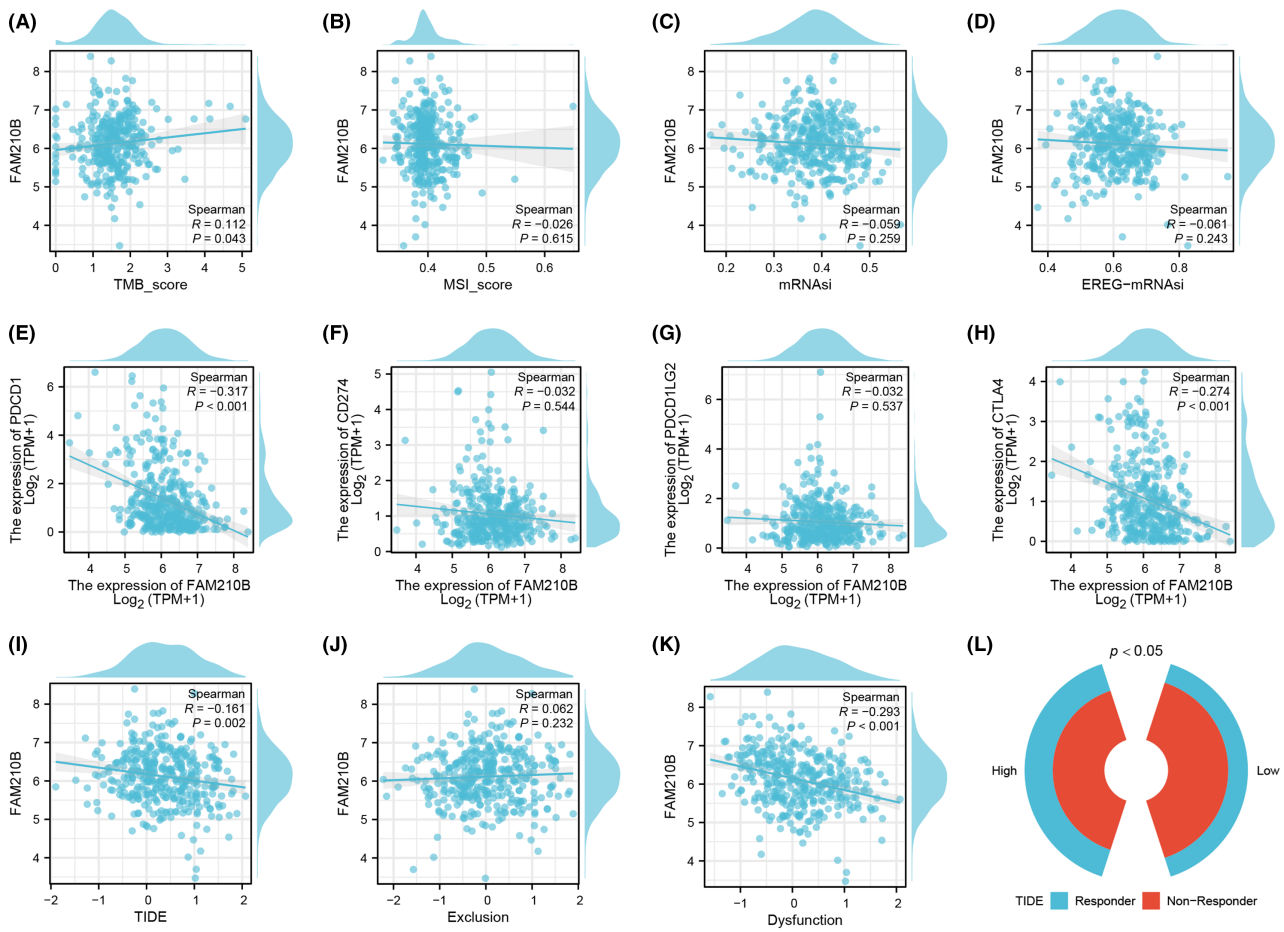
Role of FAM210B in HCC immunotherapy. (A–D): Correlation between FAM210B and tumour stemness index (TMB, MSI, mRNAsi and EREG‐mRNAsi); (E–H) Correlation between FAM210 and key immune checkpoints; (I) Correlation between TIDE and FAM210B; (J) Correlation between FAM210B and immune exclusion; (K) Correlation between FAM210B and immune dysfunction.

Exploring FAM210B's impact on immunotherapy response, we found a positive correlation with the TMB score, suggesting its association with genome instability (Figure 7A). However, no significant correlations were observed with the MSI score, mRNAsi, and EREG‐mRNAsi (Figure 7B–D). Analysis of key immune checkpoints revealed a negative correlation between FAM210B and both PDCD1 and CTLA4 (Figure 7E–H). Results of TIDE analysis showed that FAM210B was negatively correlated with TIDE score and immune dysfunction (Figure 7I–K), indicating that the patients with high FAM210B might be more sensitive to immunotherapy (Figure 7L).

### 3.7 Construction of the prognosis model based on FAM210B‐related molecules through machine learning algorithms

**FIGURE 8:**
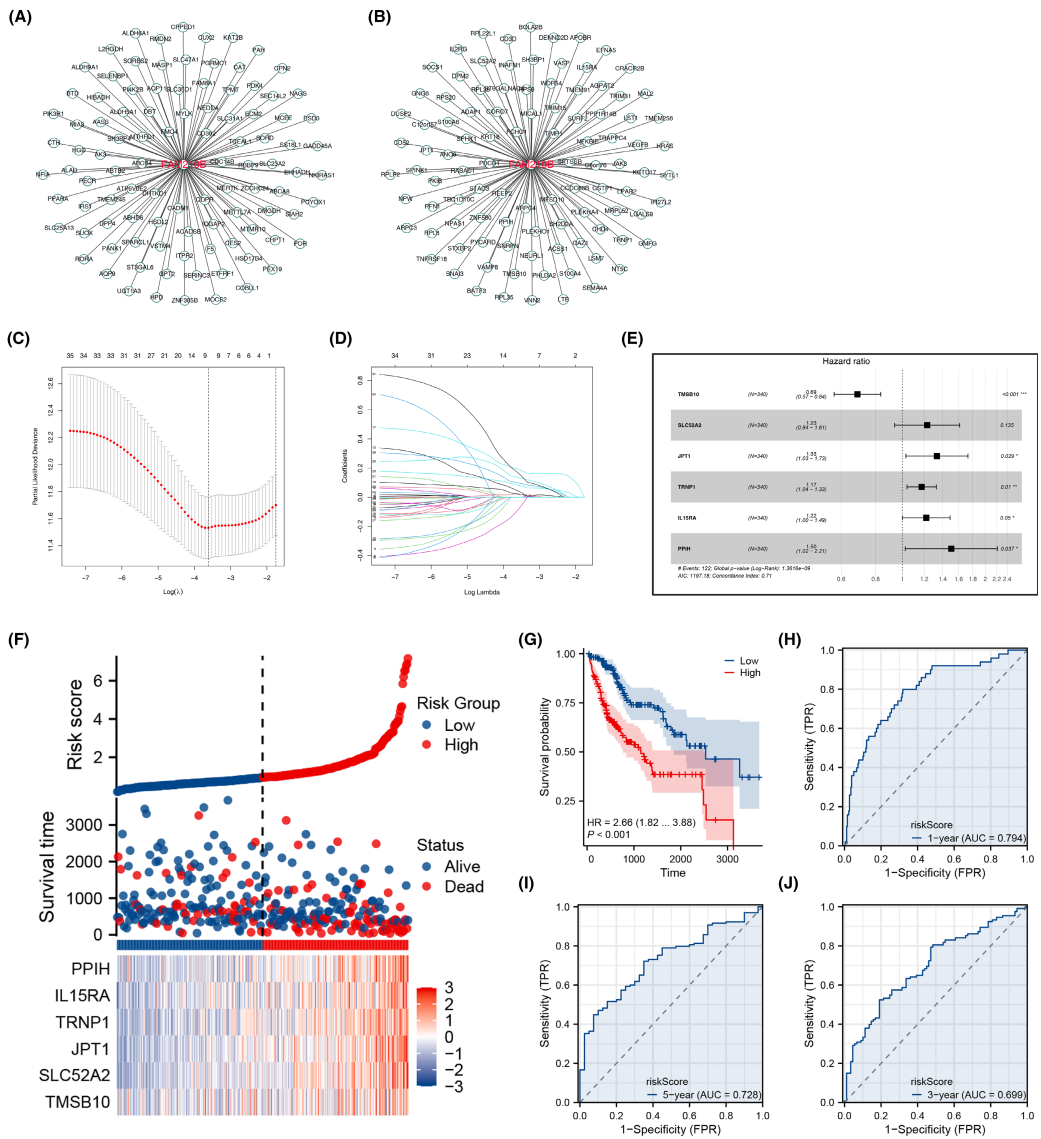
The prognosis model based on FAM210B‐related molecules through machine learning algorithms (LASSO). (A) The top 100 molecules positively correlated with FAM210B; (B) The top 100 molecules negatively correlated with FAM210B; (C, D) LASSO algorithm was used to reduce redundant variables; (E) The genes TMSB10, SLC52A2, JPT1, TRNP1, IL15RA and PPIH were identified by multivariate Cox regression analysis for the construction of prognosis model; (F) The constructed prognosis model; (G) The KM survival curves illustrated the prognosis difference between high‐ and low‐risk groups; (H–J) The ROC curves was used to evaluate the prediction ability of our model in predicting the 1‐, 3‐ and 5‐years survival.

We proceeded to ascertain molecules associated with FAM210B, as illustrated in Figure 8A,B. Initial univariate Cox regression analysis helped identify significant correlates of patient survival (Data S1). To eliminate redundancy, the LASSO regression technique was employed (Figure 8C,D). Subsequent multivariate Cox regression pinpointed critical variables for the prognostic model: TMSB10, SLC52A2, JPT1, TRNP1, IL15RA and PPIH (Figure 8E). The formulated risk score equation was: Risk score = (TMSB10 × 0.375) + (SLC52A2 × 0.207) + (JPT1 × 0.289) + (TRNP1 × 0.161) + (IL15RA × 0.199) + (PPIH × 0.409). Stratifying patients by the median risk score segregated them into the high‐risk and low‐risk groups. Notably, the high‐risk group exhibited an elevated mortality rate and poorer survival outcomes compared to the low‐risk group (Figure 8F,G; HR = 2.66, *p* < 0.001). ROC curves validated the model's efficacy in forecasting the survival at 1, 3, and 5 years (Figure 8H–J).

## 4 DISCUSSION

HCC is the leading form of liver cancer, with its prevalence highest in regions burdened by hepatitis B and C infections.^27^, ^28^ Despite treatment advances, including immunotherapy, HCC's prognosis remains poor, mainly due to late detection and the complexity of treating liver cirrhosis background.^29^ This situation underscores the importance of early diagnosis and innovative treatments, spotlighting personalized medicine's role in enhancing outcomes by addressing HCC's varied causes and molecular diversity, aiming to significantly improve survival rates.^30^

In our study, we performed a comprehensive analysis of FAM210B's expression in HCC, its biological functions, and its influence on the tumour microenvironment and treatment responses. By employing a multifaceted approach that includes pan‐cancer expression analysis, evaluation of subcellular localization, functional assays in HCC cell lines and assessments of the immune microenvironment and drug sensitivity, we seek to delineate the role of FAM210B in HCC progression and therapeutic selection. Moreover, we explore the association between FAM210B expression and key clinical outcomes, including survival, drug resistance and responsiveness to immunotherapy. Understanding FAM210B's involvement in HCC could unveil novel insights into the disease's molecular underpinnings, potentially leading to the identification of new therapeutic targets and biomarkers for personalized treatment strategies.

Our biological enrichment analysis further supports the notion that FAM210B influences key metabolic and immune response pathways in HCC. The association of high FAM210B levels with upregulated androgen response, fatty acid metabolism, and downregulated EMT and inflammatory response pathways highlights its potential role in modulating tumour biology through metabolic reprogramming and immune evasion mechanisms. The EMT pathway is a fundamental biological process that plays a crucial role in the development, tissue regeneration and pathological conditions such as cancer metastasis and fibrosis.^31^, ^32^ During EMT, epithelial cells lose their characteristic cell–cell adhesion, polarity and gain mesenchymal features, including enhanced migratory capacity, invasiveness, elevated resistance to apoptosis and greatly increased production of extracellular matrix components.^33^ In malignant tumours, EMT pathways often promote tumour progression. For example, Hua et al. found that KLK8 fosters the growth and spread of colorectal cancer by triggering the EMT process in association with PAR1 activation.^34^ Li et al. revealed that neutrophils associated with tumours trigger EMT through IL‐17a, enhancing the ability of gastric cancer cells to migrate and invade.^35^ Our biological enrichment results indicate that FAM210B may exert its tumour suppressor effect through specifically enriched biological pathways. These findings offer new avenues for exploring targeted therapies that address the metabolic and immunological vulnerabilities of HCC.

The impact of FAM210B on the HCC immune microenvironment, particularly its correlations with neutrophil infiltration and reduced B cell, memory B cell and CD8+ T cell presence, further emphasizes its role in shaping immune surveillance and response. This immune modulation may contribute to the observed associations between FAM210B expression, drug sensitivity and immunotherapy outcomes.^36^ Our data suggest that patients with high FAM210B expression might exhibit resistance to sunitinib but could benefit more from immunotherapy, implicating FAM210B as a potential biomarker for tailoring HCC treatment strategies. Moreover, the positive correlation between FAM210B and TMB highlights its possible involvement in genomic instability, a critical aspect of cancer progression and therapy resistance. The negative correlations with key immune checkpoints such as PDCD1 and CTLA4 further indicate its role in the immune escape mechanisms of HCC, providing a rationale for integrating FAM210B status into the selection criteria for immunotherapy.

Based on our findings, the therapeutic potential of FAM210B in HCC warrants further exploration. Our study reveals that FAM210B influences key metabolic and immune pathways, suggesting it could serve as both a biomarker and a target for treatment. High FAM210B levels correlate with upregulated androgen response and fatty acid metabolism, and downregulated EMT and inflammatory pathways, indicating its role in metabolic reprogramming and immune evasion. Therapeutically, targeting FAM210B could enhance the efficacy of existing treatments, particularly immunotherapy, as patients with high FAM210B expression may exhibit resistance to sunitinib but respond better to immune‐based therapies. Future research should focus on the mechanisms by which FAM210B modulates these pathways and investigate the potential of FAM210B‐targeted therapies in preclinical models. This approach could lead to personalized treatment strategies, ultimately improving patient outcomes in HCC. In discussing the limitations of our study, it's essential to acknowledge several key points that might have influenced our findings. Firstly, our data were sourced from TCGA database, primarily consisting of patient samples from Western populations. This demographic composition introduces a potential ethnic bias, as genetic and environmental factors that contribute to disease pathology can vary significantly across different populations. Consequently, the generalizability of our results to other ethnic groups may be limited, and further studies are needed to validate our findings in a more diverse cohort. Secondly, our research heavily relied on bioinformatics algorithms for data analysis. While these tools are powerful for uncovering patterns and associations within large datasets, our results might be subject to systematic biases stemming from the specific parameters or versions of R packages used. Such computational constraints could potentially influence the reproducibility and interpretation of our findings. It's important for future studies to consider these factors and, where possible, validate results using alternative computational methods or experimental approaches. Additionally, our study's sample size, derived from publicly available datasets, may limit the robustness and applicability of our conclusions. A larger, more diverse sample size would help validate our findings and provide a more comprehensive understanding of FAM210B's role in HCC. While our study utilized high‐throughput sequencing data to infer biological processes and pathways, experimental validation using in vitro and in vivo models is necessary to confirm these bioinformatics predictions.

## AUTHOR CONTRIBUTIONS

**Xianzhu Pan:** Conceptualization (equal); investigation (equal); methodology (equal); resources (equal); software (equal); visualization (equal). **Jun Xu:** Data curation (equal); investigation (equal); resources (equal); software (equal); supervision (equal); validation (equal). **Yuanqin Zhou:** Conceptualization (equal); data curation (equal); formal analysis (equal); funding acquisition (equal); software (equal); supervision (equal).

## FUNDING INFORMATION

The work is supported by the University Natural Science Research Key Project of Anhui Province (Project No. 2022AH052321).

## CONFLICT OF INTEREST STATEMENT

The authors confirm that there are no conflicts of interest.

## Supporting information


**Figure S1.** The flow chart of whole study.

**Data S1:** The results of univariate Cox regression analysis.

## Data Availability

All data are available from the corresponding author on reasonable request.
