# Supplementary figures and images for: Multifaceted role of FAM210B in hepatocellular carcinoma: Implications for tumour progression, microenvironment modulation and therapeutic selection
Source: J Cell Mol Med. 2024 Aug 28;28(16):e70031. doi: 10.1111/jcmm.70031 (PMC11358035; doi:10.1111/jcmm.70031)

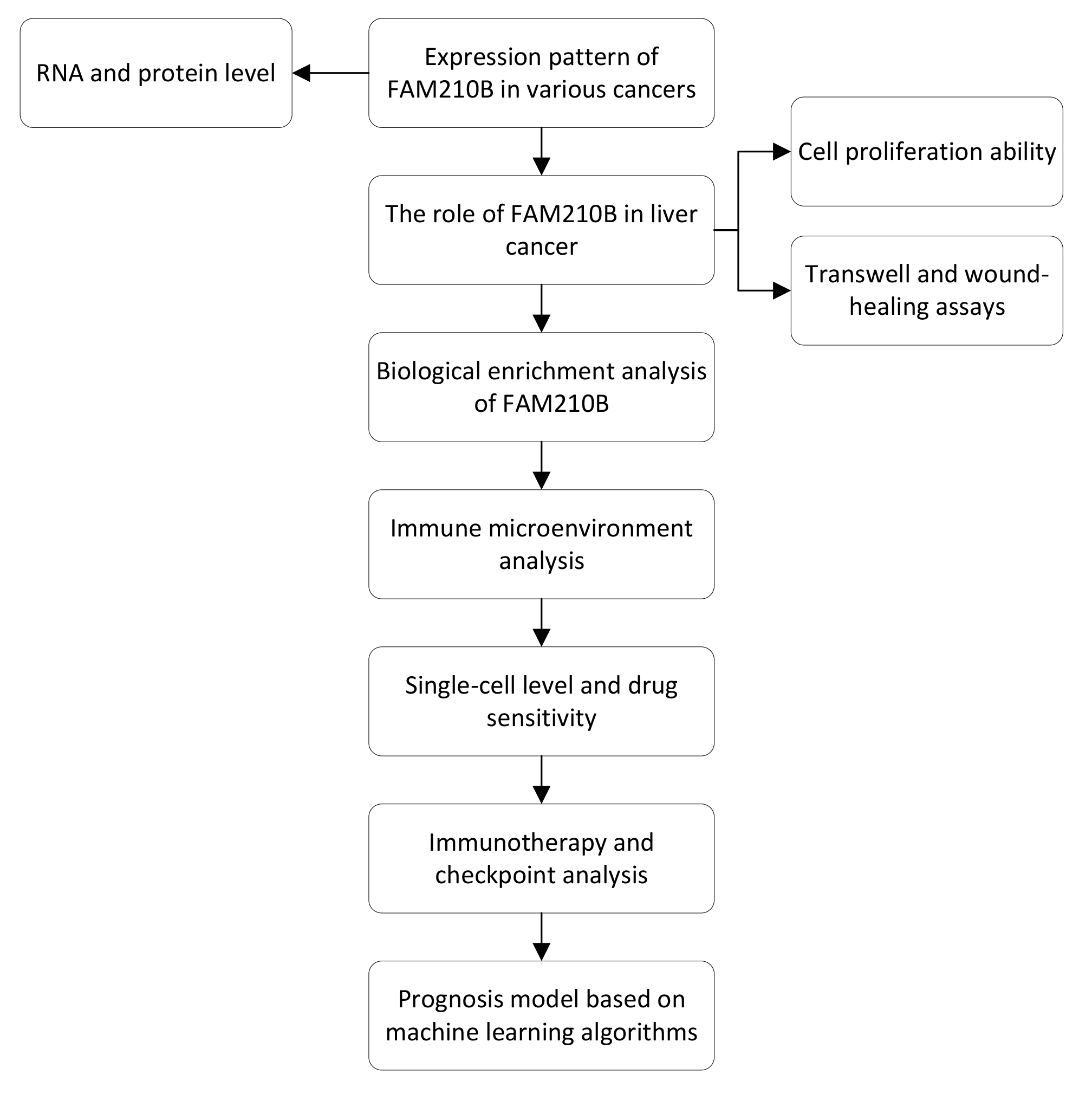

Supplement: Supplementary file 1 — Figure S1. The flow chart of whole study. [file JCMM-28-e70031-s001.tif]
